# Hughes Abdomen Closure Technique Versus Continuous Closure in Emergency Midline Laparotomy: A Randomized Controlled Study

**DOI:** 10.7759/cureus.60816

**Published:** 2024-05-21

**Authors:** Kishor Murthy, Sushanto Neogi, Sarmista Roy, Manu Vats, Rinki Meena

**Affiliations:** 1 Department of Surgery, Maulana Azad Medical College, New Delhi, IND

**Keywords:** rectus sheath closure technique, burst abdomen, midline emergency laparotomy, conventional continuous closure, hughes abdominal closure technique, abdominal dehiscence

## Abstract

Background

Abdominal wound dehiscence, a serious postoperative issue, remains a significant concern for surgeons due to its potential to increase patient mortality and morbidity. Disruption can occur at any point after surgery, sparking debate over the optimal closure method for midline vertical abdominal wounds. Therefore, it's crucial to determine the safest approach. Our randomized clinical trial is planned to compare the risk of a burst abdomen associated with the Hughes abdominal closure technique to that of continuous abdominal closure.

Methods

All patients >18 years scheduled for emergency midline laparotomy were randomly assigned into two groups using computer-generated random numbers: Group A underwent Hughes repair (12 patients) and Group B underwent continuous closure (17 patients). Preoperative data, including patient demographics, and postoperative outcomes, such as time for rectus closure, wound dehiscence, surgical site infection (SSI), and length of hospital stay, were documented for analysis.

Results

The study found that the average patient age was 37.89 years, with more males than females. Both groups had an equal distribution of co-morbidities (p = 0.468), but none of these factors were statistically significant. Burst abdomen occurred in 25% of group A and 41.1% of group B (p = 0.367, not significant). Incisional hernia was absent in both groups. Surgical site infection (p = 0.119) and respiratory complications (p = 0.16) were not statistically significant between groups. However, in group A, the regressive analysis showed significant associations between burst abdomen, surgical site infection (p = 0.018), and respiratory complications (p = 0.007), while in group B, these associations were not significant (p = 0.252 for SSI and p = 0.906 for respiratory complications).

Conclusion

The occurrence of burst abdomen and closure time differences between continuous and Hughes techniques were not significant. The Hughes technique was quicker to learn (32 vs. 22 minutes). Burst abdomen was more common in continuous closure (group A: 25% vs. group B: 41%), favoring the Hughes technique. Factors like age, gender, and others didn't significantly impact the burst abdomen in either group.

## Introduction

Abdominal dehiscence is the disruption or break of a wound with the separation of the musculoaponeurotic layer [1,2]. It can be partial or complete. Abdominal wound dehiscence (burst abdomen, fascial dehiscence) is a severe postoperative complication that continues to plague surgeons and threatens the lives of patients with increased mortality and morbidity. In India, incidence ranges from 10% to 30% and results from failure of the deeper portions of the abdominal incision to unite, resulting in protrusion of abdominal contents, usually bowel, through the disrupted wound [3]. At a later stage, it may appear as an incisional hernia. Significant wound dehiscence occurs in approximately 1% of all laparotomies [4]. The incidence of wound disruption increases with the presence of predisposing factors.

There have been several studies evaluating various closure techniques. There is a debate about the best method of closure of a midline vertical abdominal wound following an emergency laparotomy [4]. Studies in Western countries have found no significant difference in the risk of burst between continuous and interrupted methods [5]. Hence it is imperative for us to ascertain the safest method of closing the abdomen. The present randomized clinical trial is planned to compare the risk of a burst abdomen with Hughes's abdominal closure technique to that of continuous abdominal closure.

## Materials and methods

This randomized controlled study was conducted in a tertiary care hospital in the Department of Surgery, Maulana Azad Medical College, Lok Nayak Hospital and associated hospitals, New Delhi, from February 2021 to March 2022 after institutional scientific committee and ethics committee approval.

Study population

All patients aged >18 years scheduled for midline laparotomy for surgical emergency presenting at Lok Nayak Hospital between February 2021 and March 2022 were included in this study. Patients who underwent previous laparotomies have severe anemia (Hb <7g%), uncontrolled diabetes mellitus, jaundice, immunocompromised states, chronic renal failure, pregnancy and lactation (duration <6 m) were excluded from this study.

Sample size

According to Bansiwal et al. [6] study, the incidence of abdominal wound dehiscence in emergency laparotomy with continuous closure was 20.1% and that of interrupted closure technique (since Hughes repair is also a type of interrupted closure) was 5.4%. We used the formula below to calculate the sample size.

N = [Z_1-α/2_ √(2{P(1-P)}) + Z_1-β_√(P_1_(1-P_1_)+P_2_(1-P_2_))]^2^/(P_1_-P_2_)^2^

Using this in a formula with 80% power, 95% confidence level, and 5% α error, where P_1 _= 20.1%, P_2 _= 5.4%, P = (P_1 _+ P_2_)/2, Z_1-α/2 _= 1.96, Z_1-β _= 0.84, which makes sample size required N = 84.85. As per the formula, the cases required are 85, but due to the time constraint of the study and the ongoing COVID-19 pandemic a minimum sample of 20 with 10 in each group was taken. Initially, we had committed 10 patients in each group (20 total), but with our hospital opening after the COVID-19 enforced break, we could enroll a total of 29 patients in the study. They were randomized into two groups (group A and group B) using a computer-generated random number table, with group A having 12 and group b having 17 patients. Written and informed consent was obtained from patients. Patients were given the option to opt out of the study at any time, with confidentiality and privacy being ensured at all stages of the study period. The collected data were used for study purposes only.

Methodology 

**Figure 1:**
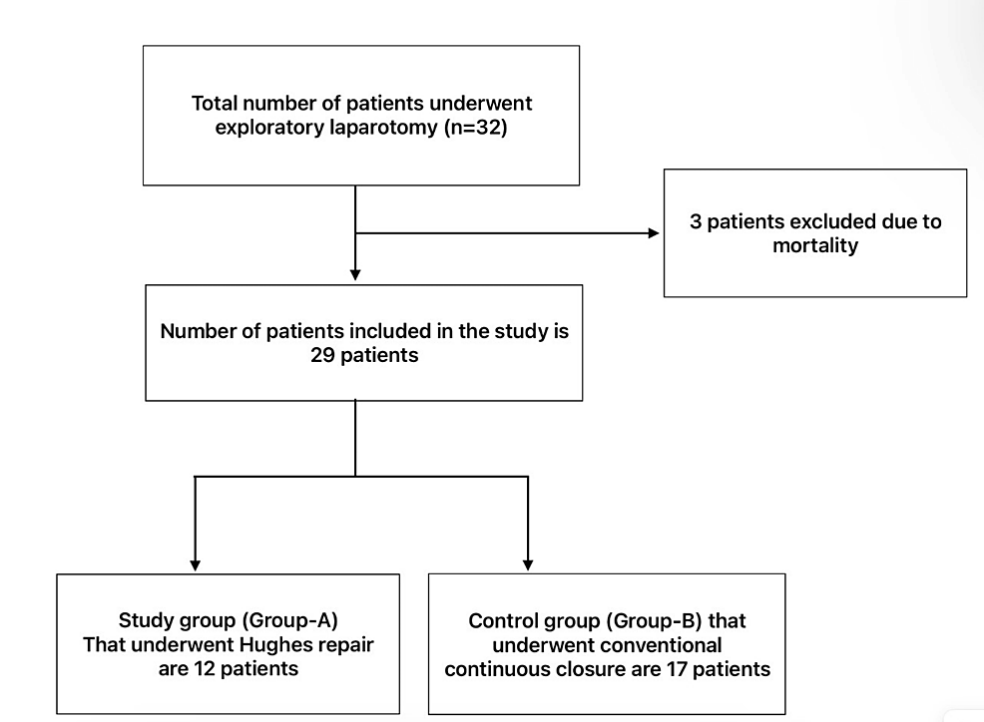
Flow chart of the study.

Patients undergoing emergency exploratory laparotomy surgery from February 2021 to March 2022 who fulfilled the inclusion criteria were approached after a complete history, examination, radiological and biochemical tests were performed, and informed consent was obtained. Patients were randomized into two groups by computer-generated random numbers. Figure 1 explains the course of our study. In our study, three mortalities were excluded due to septic shock on postoperative day one, and two others due to dyselectrolytemia on postoperative days one and two, respectively.

Broad-spectrum antibiotics in combination with Augmentin, Gentamicin, and Metronidazole were given at the time of induction of anesthesia and continued for seven days. Patients were followed up for routine postoperative care, and laparotomy wounds were inspected daily for any redness, discharge from wound, swelling of wound, gaping, or visible gut contents. Risk predictors of wound dehiscence, such as chronic respiratory conditions causing cough, hypoalbuminemia, obesity, age, and sex, were noted.

Operational definitions

Group A-Hughes Abdomen Repair Technique

**Figure 2:**
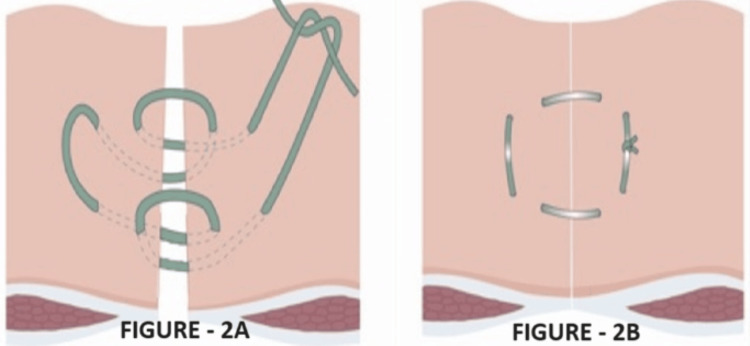
Hughes closure (A) shows suture placement and (B) shows repair after closure. Diagram showing the Hughes closure using a series of horizontal and two vertical mattress sutures within a single suture. When the sutures are pulled to close the defect, the sutures lie both across and along the incision. Source: Cornish et al. Hughes Abdominal Repair Trial (HART) [7].

Starting 2 cm from the edge of the linea, a stitch is made outside-in, followed by a 0.5 cm stitch inside-out on the opposite side. This pattern continues with near and far stitches alternately on each side to form a horizontal mattress suture. Finally, the suture ends are tied to bring the linea alba edges together, as shown in Figure 2.

Group B: Continuous Closure

Suture bites will be placed 1 cm from the cut edge of linea alba and successive bites will be taken 1 cm from each other. The edges of linea alba will be gently approximated by keeping a suture-to-wound length ratio of 4:1.

Statistical analysis

Data was entered, compiled, and processed in Microsoft Excel (Microsoft Corporation, Redmond, Washington, USA) and SPSS version 25.0 (IBM Corp., Armonk, New York, USA). For qualitative data, the Chi-square test was used, and for quantitative data, the student t-test was used, and the data were expressed as the mean and SD of the difference between two means for paired observation. A P-value <0.05 was considered statistically significant.

## Results

Table 1 shows the general patient characteristics of both groups.

**Table 1:** Patient characteristics and co-morbidities. COPD: chronic obstructive pulmonary disease.

Parameters	Group A (12 patients)	Group B (17 patients)
Age (years)	41.25	34.53
Male:female	11:1	12:5
Hemoglobin <10 g/dl	3 (25%)	8 (47.05%)
Total leucocyte count (<4000 or >11,000 cells/mm^3^	5 (41.6%)	11 (64.7%)
Serum albumin (<3 g/dl)	4 (33.3%)	11 (64.7%)
COPD	0 (0%)	2 (11.76%)
Hypertension	0 (0%)	2 (11.76%)
Type 2 diabetes mellitus	1 (8.33%)	0 (0%)

Burst abdomen had an equal distribution in group A of 25% and group B had 41.1% with a p-value of 0.367, which is not significant. Surgical site infection (SSI) incidence and respiratory complications have p-values of 0.119 and 0.16, respectively, and are statistically insignificant. An incisional hernia was not seen in both groups. Table 2 compares the patient outcomes between the two groups.

**Table 2:** Patient outcomes.

Parameters	Group A (12 patients)	Group B (17 patients)	P-value
Wound dehiscence	3 (25%)	7 (41.1%)	0.367
Day of wound dehiscence	5	4.57	0.622
Surgical site infection	5 (41.6%)	12 (70.5%)	0.116
Respiratory complications	2 (16.6%)	7 (41.1%)	0.16
Days of hospital stay	10.58	15	0.301
Length of incision (cm)	15.17	16	0.094
Duration of rectus closure (mins)	26.08	24.24	0.068
Incisional hernia	0	0	
Fever	4	8	0.296

On regressive analysis of burst abdomen with other parameters, surgical site infection and respiratory complications with p-values of 0.018 and 0.007 are significant in group A, whereas, in group B, they are not significant, with p-values of 0.252 (SSI) and 0.906 (respiratory complications). Regression analysis of burst abdomen and other complications is shown in Table 3.

**Table 3:** Regression analysis for burst abdomen.

Parameters	Group A (P-value)	Group B (P-value)
Age (years)	0.864	0.464
Gender	0.546	0.949
Hemoglobin (<10 g/Dl)	0.07	0.9
Total leucocyte count (<4000-11,000 cells/cumm)	0.7	0.906
Respiratory complications	0.007	0.906
Random blood sugars	0.682	0.594
Serum albumin	0.83	0.36
Duration of disease	0.36	0.239
Surgical site infection	0.018	0.252

## Discussion

The primary cause of acute abdomen in 70-80% of patients without trauma is perforation peritonitis. Postoperative wound dehiscence presents a significant concern for surgeons, with an incidence rate ranging from 1 to 3%. Patient-related factors contributing to this risk include extremes of age, anemia, uremia, jaundice, diabetes mellitus, hypoalbuminemia, deficiencies in zinc and vitamin C, and the use of medications such as steroids, anti-neoplastic drugs, and radiation. Surgeon-related factors primarily involve the technique used for rectus sheath closure, with faulty closure being a major contributor [1].

The mean time required for rectus closure was 24.16 minutes, with group A averaging 26.08 minutes and group B averaging 24.24 minutes. Although the difference between the groups was not statistically significant (p-value 0.068), other studies by Sharma et al. and Shashikala et al. showed statistically significant results regarding the time taken for rectus closure [8,9]. In our study, 34.5% of patients experienced wound dehiscence out of a total of 10 patients. Out of 23 patients, 25% in group A and 41.2% in group B experienced wound dehiscence. However, this disparity was not statistically significant (p-value 0.367). The study by Sharma et al. (20%), Kumar et al. and Gurjar et al. found no difference between both the suture technique groups [8,10,11].

Despite the lack of statistical significance in our data, the occurrence of burst abdomen was higher in the continuous closure group (25% in group A vs. 41% in group B). Considering the smaller sample size in both groups, a larger sample size might have yielded significant results. In our study, the average day of wound dehiscence was 4.78 days, ranging from three to six days. This contrasts with documented ranges in the literature, like Gupta et al., which typically extend from the sixth to the ninth day [12]. Early occurrences of burst abdomens in our cases may be attributed to delayed presentation, pyoperitoneum, and poor nutritional status.

Surgical site infection was observed in 58.6% of patients in this study, with no significant difference between the groups (p-value 0.367). Studies by Kumar et al. and Al-Faouri et al. did not find any statistical significance among groups [10,13]. However, studies by Agrawal et al. and Dhamnaskar et al. were statistically significant [14,15]. Regression analysis revealed a statistically significant association with wound dehiscence of group A (p-value 0.018), but not group B (p-value 0.252).

In our study, 31% had respiratory complications like pleural effusion, consolidation and lung atelectasis with a non-significant p-value of 0.16. Other studies reported incidences of burst abdomen: Jaiswal et al. (52.4%) and Mehdorn et al. (9%) [16,17]. Regression analysis with burst abdomen showed a significant p-value of 0.007 in group A but a non-significant p-value of 0.906 in group B. Respiratory complications during the postoperative period, marked by coughing and heightened use of accessory muscles, lead to elevated intra-abdominal pressure. During the six-month follow-up for incisional hernia, no cases were reported in either group following clinical and ultrasonographic evaluation. However, there are limited studies available to compare incisional hernia rates in the short term.

Based on the findings, the Hughes technique emerges as a favorable surgical approach for rectus sheath closure in midline laparotomy. It is efficient, does not prolong anesthesia duration significantly, and can be quickly learned and implemented. For patients with delayed presentation of perforation peritonitis and substantial pyoperitoneum, utilizing the Hughes technique for rectus sheath closure is recommended to reduce the risk of postoperative wound dehiscence.

Limitations

The study was conducted with a relatively small sample size at a single center, which limit the findings' generalizability.

## Conclusions

Surgical technique emerges as the foremost predictor of wound dehiscence, with respiratory complications and surgical site infections also playing pivotal roles. While the incidence of burst abdomen did not reach statistical significance, there was a notable disparity in percentages between the Hughes technique (group A: 25%) and continuous closure (group B: 41%), suggesting the superiority of the Hughes technique for rectus closure. Moreover, the mean duration for continuous closure versus the Hughes technique showed no statistical variance, indicating the ease of learning the Hughes technique despite a slight difference in time (32 minutes compared to 22 minutes). When analyzing various factors influencing burst abdomen, such as age, gender, serum albumin, total leukocyte count, hemoglobin levels, and duration of stay, none exhibited a significant association with burst abdomen in either group.
